# Uncharted features and dynamics of reading: Voices, characters, and crossing of experiences

**DOI:** 10.1016/j.concog.2017.01.003

**Published:** 2017-03

**Authors:** Ben Alderson-Day, Marco Bernini, Charles Fernyhough

**Affiliations:** a Department of Psychology, Durham University, Science Laboratories, South Road, Durham DH1 3LE, United Kingdom; b Department of English Studies, Durham University, Hallgarth House, 77 Hallgarth Street, Durham DH1 3AY, United Kingdom

**Keywords:** Inner speech, Imagery, Hallucinations, Theory-of-mind, Creativity

## Abstract

Readers often describe vivid experiences of voices and characters in a manner that has been likened to hallucination. Little is known, however, of how common such experiences are, nor the individual differences they may reflect. Here we present the results of a 2014 survey conducted in collaboration with a national UK newspaper and an international book festival. Participants (*n* = 1566) completed measures of reading imagery, inner speech, and hallucination-proneness, including 413 participants who provided detailed free-text descriptions of their reading experiences. Hierarchical regression analysis indicated that reading imagery was related to phenomenological characteristics of inner speech and proneness to hallucination-like experiences. However, qualitative analysis of reader’s accounts suggested that vivid reading experiences were marked not just by auditory phenomenology, but also their tendency to cross over into non-reading contexts. This supports social-cognitive accounts of reading while highlighting a role for involuntary and uncontrolled personality models in the experience of fictional characters.

## Highlights

• Vivid experiences of characters implicate simulation processes during reading.
• Reading imagery is related to features of inner speech and hallucination-proneness.
• Qualitative analysis highlights involuntary experiences of characters and voices.

## 1 Introduction

Vivid or immersive experiences are often described in relation to reading fictional narratives (Caracciolo and Hurlburt, 2016, Green, 2004, Ryan, 1999, Ryan, 2015). In particular, it seems common for readers (and writers) to report “hearing” the voices of fictional characters, in a way that suggests they have a life of their own (Vilhauer, 2016, Waugh, 2015). What, though, does this mean for the psychological processes that may underpin such experiences? Psychological studies on the phenomenological experience of reading have tended to focus on two strands: first, the perceptual and sensory qualities of reading – primarily via notions of ‘voice’ and inner speech (Alexander and Nygaard, 2008, Perrone-Bertolotti et al., 2014); and second, how the reader represents the characters and agents of a text (Kidd and Castano, 2013, Mar and Oatley, 2008).

To a certain extent, it is intuitive to understand why a text – even if not read out loud – would need to be voiced in some way to be read. This is sometimes conceptualized either as inner speech – namely, the various ways in which people talk to themselves (Alderson-Day & Fernyhough, 2015) – or more broadly in terms of auditory imagery, i.e. purposefully imagining the qualities of characters’ or narrators’ voices (Hubbard, 2010, Kuzmičová, 2013). Evidence of inner speech involvement comes from psycholinguistic studies on reading: when we read, phonologically longer stimuli take longer to read than shorter stimuli of the same orthographic length (Abramson and Goldinger, 1997, Smith et al., 1992), while acoustic properties of one’s own voice, such as accent, can affect our expectation of rhyme and prosody (e.g., Filik & Barber, 2011). This suggests that at least some properties of text are sounded out in inner speech during reading (Ehrich, 2006).

Readers’ expectations of character and narrator voices can also affect how a text is processed. For instance, readers adjust their reading times for texts written by authors with a slow or fast-paced voice. People reading difficult texts, and those who report more vivid mental imagery, show greater evidence of such “author voice” effects on reading speed (Alexander & Nygaard, 2008). When characters’ words are referred to in direct speech, voice-selective regions of auditory cortex are more active than during indirect reference (e.g., ‘He said, “I hate that cat”’ vs. ‘He said that he hates that cat’), suggesting auditory simulation of character’s voices (Yao, Belin, & Scheepers, 2011). Evidence of inner speech and auditory imagery being involved in reading is consistent with broader theories of reading that place perceptual simulation and embodiment at the heart of textual comprehension (Zwaan, 2004, Zwaan et al., 2004), i.e. the idea that sensorimotor imagery processes are automatically engaged when we read text, as part of understanding the meaning of what is being described.

A second strand of research has emphasized the role of social cognition in the reading experience, largely in response to literary fictional texts. Many readers strongly personify characters and narrators by making inferences about their described thoughts and behaviors (Bortolussi & Dixon, 2003) and assigning them intentionality (Herman, 2008). Studies on empathy in literary experiences have focused on how empathetic engagement with characters is triggered by specific discourse strategies (e.g., first-person vs. third-person narratives; Keen, 2006) or on how empathy in the act of reading relies on readers’ previous personal experiences (Kuiken et al., 2004, Miall, 2011). Other psychological approaches to reading have investigated the “projection” of knowledge that readers perform – the process by which they assign to each character an individual epistemic view of the narrative world (Gerrig, Brennan, & Ohaeri, 2001), which allows for narrative dynamics such as “suspense” (Gerrig, 1989). Based on such processes, it has been argued that the ways in which readers attribute consciousness, mental states, intentions, and beliefs to characters recruits (Zunshine, 2006, Zunshine, 2012) or even enhances (Kidd & Castano, 2013) readers’ theory-of-mind, i.e. the ability to represent the mental states of others. Indeed, it has been claimed that the “function” of reading fiction may be to simulate social experiences involving other people (Mar & Oatley, 2008).

Taken together, the above studies highlight some of the separate perceptual and social-cognitive processes that could explain accounts of ‘hearing’ the voices of characters. But, although the experiences they are based on are intuitively familiar, phenomenological data on the reading experience in the words of readers themselves is surprisingly lacking. Indeed, almost all of the above work has involved either experimental manipulation of texts, or analysis of responses to specific literary texts. Systematic surveys of readers’ experiences of characters in general – that is, as part of their day-to-day experience of reading for pleasure – are largely absent.

We know of only one recent exception: Vilhauer (2016) conducted a qualitative analysis of 160 posts that resulted from a search of ‘hearing voices’ and ‘reading’ from a popular message-board website (*Yahoo! Answers).* Of these, over 80% reported vivid experiences when reading, the majority describing specific auditory properties including volume, pitch, and tone. Qualities that are perhaps more indicative of social representation – such as identity and control – were also reported in some cases, but were often hard to classify or lacking in detail. However, the open-ended structure of the source material used by Vilhauer (2016), the fact that it was gathered based on the specific keywords ‘hearing voices’, and the lack of demographic data from the study participants limit any strong generalizations about the reading experience.

As such, it is unclear whether vivid examples of characters’ voices – or indeed other kinds of character representation – are actually a common part of the reading experience. It could be that the act of reading about characters simply involves combining such features in an additive and largely automatic way: if so, phenomenological reports may be expected to consist of vivid perceptual imagery plus some kind of mental state representation – a clear experience of a character’s voice and their emotional state, for example. But such skills also vary considerably in the general population (Isaac and Marks, 1994, Palmer et al., 2003) and may not be integral for most people, most of the time: for some, experiences of voices, characters, or other features of a text could combine to create something very different entirely, or even nothing at all (a character’s voice without any impression of intentionality, for example).

To investigate this question, we collaborated with the Edinburgh International Book Festival and a national UK newspaper (the *Guardian*), to survey a large sample of readers about their inner experiences. Instead of focusing on the experience of a particular text (e.g., Miall & Kuiken, 1999), or experimentally varying textual properties (Dixon & Bortolussi, 1996), we opted for a general questionnaire about readers’ encounters with voices and characters. This encompassed all kinds of reading (prose vs poetry; crime fiction vs historical novels; or fictional vs non-fictional narratives), although the large majority of eventual responses related to engaging with fiction (82%).

The first aim of the survey was to gather quantitative information on the vividness of readers’ experiences, and examine how that related to other individual differences in potentially similar processes. Based on the putative involvement of inner speech in reading, we included a measure of everyday inner speech experiences: the Varieties of Inner Speech Questionnaire (VISQ: McCarthy-Jones & Fernyhough, 2011). Derived from developmental theories of self-talk (Vygotsky, 1987), the VISQ measures a range of phenomenal properties of inner speech, including the extent to which it includes dialogue, if it is experienced in full sentences, whether it is evaluative or motivating, and whether it includes other people’s voices. If readers were more likely to report vivid experiences of voice and character during reading, they might also be expected to have a more vivid experience of their own inner speech in general. In addition, vivid experiences of voices and characters during reading have been likened by some (e.g., Vilhauer, 2016) to be similar to actual experiences of ‘hearing voices’ or auditory verbal hallucinations (AVHs). Although direct parallels with florid and distressing experiences are unlikely, traits towards having unusually vivid and hallucination-like experiences are thought to exist along a continuum in the general population (Johns & van Os, 2001). They may, therefore, relate to reports of particularly vivid reading experiences. To investigate this further, we included a short measure of auditory hallucination-proneness (the Launay-Slade Hallucination Scale – Revised; Bentall & Slade, 1985). We predicted that participants with more vivid experiences of reading in general would also be more prone to hallucination-like experiences.

Our second aim was to qualitatively explore readers’ own descriptions of voice and character, via a free-text section of the survey. Such descriptions offer a nuanced and detailed picture of the inner experience of reading that might otherwise be lost in purely quantitative approaches to the topic. Many of the previously mentioned studies have focused on a very specific aspect of reading (activation of inner speech, auditory imagery, mental imagery, empathy for characters, projection of knowledge, etc.) without attempting a unified account of how all these aspects relate to each other and trigger other, richer processes. To address this, readers’ descriptions in our study were coded in terms of representational *features* of the experience (such as the different sensory modalities involved), but also their *dynamics*, namely the processes by which the experiences seemed to occur. While this analysis was primarily driven by the main themes apparent in the data, our descriptions in some cases required the creation of new terms or their importing from narratological and linguistic research on fictional narratives. For example, here we have used the term “mindstyle” – a concept borrowed from linguistics (Fowler, 1977, McIntyre and Archer, 2010, Semino, 2007) – to refer to the unique way in which a person or character thinks about and views the world, and an idea that is clearly relevant for social simulation accounts of the reading experience. This and other terms used in the coding are expanded on below.

## 2 Methods and materials

### 2.1 Participants

Participants were invited to take part in an online survey on readers’ inner voices via a series of blogposts for the Books and Science sections of the *Guardian* website (‘Inner Voices’), publicity at the Edinburgh International Book Festival (EIBF) 2014, social media, and a project website (www.hearingthevoice.org). A total of 1566 participants (75% F/24% M/1% Other; Age *M* = 38.85y, *SD* = 13.48y, Range 18–81) took part in the survey, with responses primarily coming from English-speaking countries (UK, USA, Australia, Ireland, and Canada; see Table 1 for demographic details). Participants were also asked to indicate their level of education and their general reading preferences. This indicated that the sample had a high level of educational achievement on average (over 80% possessing a graduate degree or higher), with the most popular reading preferences being for general fiction, literary classics, and historical fiction. The survey was live for 6 weeks, and all procedures were approved by a local university ethics committee.

### 2.2 Measures

The survey was divided into two parts. Section 1 – the Readers’ Imagery Questionnaire – specifically asked about participants’ vivid experiences of voices and characters during reading. Section 2 included the questionnaire items on inner speech and auditory hallucination proneness.

#### 2.2.1 Reading imagery questionnaire

A reading imagery questionnaire was devised specifically for the present study based on commonly used measures of imagery, including Betts’ Questionnaire upon Mental Imagery (Sheehan, 1967) and the Vividness of Visual Imagery Questionnaire (Marks, 1973). It consisted of five items, each answered on a 5-point Likert scale (see Table 2):

(i) Do you ever hear characters’ voices when you are reading?
(ii) Do you have visual or other sensory experiences of characters when reading?
(iii) How easy do you find it to imagine a character’s voice when reading?
(iv) How vivid are characters’ voices when you read?
(v) Do you ever experience the voices of characters when not reading?

To elicit more phenomenological detail about the experience, a further question asked: ‘If you feel that you have had particularly vivid experiences of characters' voices, please describe them in the box below’. Responses up to a 500-word limit were allowed. Qualitative analysis was then conducted on the readers’ responses to this question specifically (see below).

#### 2.2.2 Varieties of inner speech questionnaire (VISQ: McCarthy-Jones & Fernyhough, 2011)

The VISQ includes 18 items on the phenomenological properties of inner speech. It includes four subscales: *dialogic inner speech* (e.g., ‘I talk back and forward to myself in my mind about things’); *evaluative/motivational inner speech* (‘I think in inner speech about what I have done, and whether it was right or not’); *other people in inner speech* (‘I hear other people’s voices nagging me in my head’); and *condensed inner speech* (‘I think to myself in words using brief phrases and single words rather than full sentences’). Participants rated their agreement with these statements on a 6-point Likert scale ranging from “Certainly does not apply to me” to “Certainly applies to me”. Each subscale has good internal and test-retest reliability (Alderson-Day et al., 2014, McCarthy-Jones and Fernyhough, 2011).

#### 2.2.3 Launay-Slade hallucination scale – revised (LSHS: Bentall & Slade, 1985)

A short version of the LSHS was used to assess susceptibility to auditory hallucinations. Five items that specifically related to unusual auditory experiences were selected from the Revised Launay Slade Hallucination Scale used in Morrison, Wells, and Nothard (2000), for example: ‘I hear people call my name and find that nobody has done so’. Participants indicate their agreement on a 4-point scale ranging from ‘Never’ (1) to ‘Almost Always’ (4). Data on the 5-item version reported by McCarthy-Jones and Fernyhough (2011) and Alderson-Day et al. (2014) have shown the scale to have moderate/good internal reliability (Cronbach’s alpha >0.69).

As part of a separate study, participants also completed questionnaires on inner speech frequency and imaginary companions. While this will be fully reported elsewhere, here we have included some preliminary data on inner speech frequency that corroborates the other, agreement-based VISQ results (see Footnote 2).

### 2.3 Quantitative analysis & qualitative coding

A mixed methods approach was used to (i) analyse the relations between questionnaire measures collected in Sections 1, 2, and (ii) qualitatively code free-text responses given at the end of the Readers’ Imagery Questionnaire. Questionnaire answers to Sections 1, 2 were analysed using Spearman’s Rho correlation co-efficients (due to non-normal distributions in some of the questionnaire outcomes) and hierarchical regression analysis, using total score for reading imagery as the dependent variable. A Bonferroni correction was applied to all pairwise correlations tested to avoid inflated type 1 error incurred from multiple comparisons. Missing questionnaire responses were replaced with the mean per item.

Free-text responses from Section 1 were coded using an inductive thematic analysis (Braun & Clarke, 2006). Two raters (BA-D and MB) first independently devised a series of descriptive codes from the entire dataset. Codes were then discussed, refined, and applied to 20% of the dataset for parallel coding by each rater, before independent coding of the remainder. Inter-rater reliability for coding was high (*k* = 0.82). The coding scheme classified answers in two ways: firstly, for their general *features*, including presence of specific sensory properties; and secondly in terms of *dynamics*, or descriptions of the overall experience and imaginative process described by the reader. Although each response could only receive each code once (e.g., descriptions of experiences with multiple visual features nevertheless only received one visual code), any given answer could be classed as having several different features and dynamics. Along with the term “mindstyle”*,* our novel codes included “blending”, a term used by Fauconnier and Turner (2003) to denote the mixing of concepts from multiple domains to form new combinations (as in, for example, the creation of novel imagery). In contrast, “experiential crossing” is a new term that we use here to refer to instances of voices and characters being experienced beyond the immediate context of reading. A full list of codes is provided in Table 3. Unless otherwise indicated, all italicization in example quotes has been added by the authors to illustrate how specific content relates to specific codes allocated. For clarity, all references to book titles have been placed in inverted commas.

## 3 Results

### 3.1 Readers’ experiences of voices and characters: summary data

Less than 1% of responses were left blank in Section 1 (the Reading Imagery Questionnaire; RIQ). Table 2 shows the frequency of response for each of the questions on vivid reading experiences. Most participants reported hearing characters’ voices when reading at least some of the time, with over half (51%) hearing them most or all of the time (Q1). Visual and other sensory experiences were endorsed to a similar degree (Q2). Approximately two thirds of participants described it as being fairly or very easy to imagine characters’ voices when reading (Q3), while the vividness of voices varied from being vaguely present to being as vivid as listening to an actual person (Q4). Experience of characters’ voices outside of reading was, in contrast, relatively rare: a fifth of participants described this experience happening sometimes, but less than 4% of participants reported this happening most or all of the time (Q5).

### 3.2 Relations between reading experiences, inner speech, and auditory hallucination-proneness

Within the sample, 1522 participants also completed measures of inner speech (the VISQ) and hallucination-proneness (the LSHS). Less than 3% of responses were left blank. As the internal reliability of the five RIQ items was good (Cronbach’s alpha = 0.80), scores across the five items were summed to provide a total score of readers’ susceptibility to vivid reading experiences.

Correlational analysis indicated that total score on the RIQ was positively related to auditory hallucination-proneness (*r* = 0.25), other people in inner speech (*r* = 0.38), dialogic inner speech (*r* = 0.23), and evaluative/motivational inner speech (*r* = 0.20, all *p* < 0.001, Bonferroni corrected). Reading scores also negatively correlated with condensed inner speech (*r* = −0.13), i.e. participants with more expanded than condensed inner speech also had more vivid reading experiences.

When these factors were assessed in a hierarchical regression model, only a subset of predictors was retained (see Table 4). Using total score on the reading items as the dependent variable, Age, Gender,^2^ and Education Level were included as control predictors in block 1, followed by each of the VISQ subscales and total score on the LSHS. The final model significantly predicted total RIQ score (*F* = 43.06, *p* < 0.001) accounting for 18% of the variance. Significant predictors retained by the model were: LSHS (*p* < 0.001, **β** = 0.11); dialogic inner speech (*p* < 0.001, **β** = 0.11); other people in inner speech (*p* < 0.001, **β** = 0.30); and condensed inner speech (*p* < 0.001, **β** = −0.09). Age, gender, education level, and evaluative inner speech were all non-significant (gender: *p* = 0.08, all other *p* > 0.50).^3^

### 3.3 Reader’s experiences: detailed qualitative descriptions

Further description of reading experiences was provided by 413 participants. Free text responses ranged from 1- to 320-word answers (answer length *M* = 41.71 words, *SD* = 35.26 words).

#### 3.3.1 Features

As may be expected, descriptions of hearing characters’ voices and references to specific characters were very common, occurring in 291 cases (70%). Many participants reported a strong and vivid engagement with the characters of a text. For example, one participant stated [emphasis added throughout]:

I become so engrossed in a novel that the characters become real to me. I know that they are not real, but *they feel real*. It is as vivid as watching the characters in a film on TV where the screen is my mind's eye. In fact, *if I can't hear the characters' voices, I find it impossible to carry on reading the novel* because it is the vivid experience of characters' voices (or sometimes the author's “voice”) that I want when I read a novel.

Characters, however, were not the only voices present, despite the questionnaire focusing on characters specifically. 49 participants (12%) described vivid perceptions of the author and/or narrator’s voice in a text. For example:

There are times when reading, that the narrative draws me in such that the words are no longer words just the world they describe. But in a few cases, *a narrator’s voice comes through, almost as if one was listening to someone reading it* […]

Well composed dialogue often creates *a sense of 'listening' to a real conversation with varied tones and accents* for me. I can also feel as if a strong first person narrative voice is *“speaking” fully to me* […].

Auditory characteristics of voices were reported in the large majority of cases. In 80 cases (19%) this involved the specific mention of accents without any reference to other auditory properties:

I can hear *male and female, young and old, accents that I can't speak myself* [….]

When I read books whose characters are English, Scottish, Irish, or any of the strong American dialects*, I hear them speaking with that dialect* – even without relying on the author's use of it in writing what they say.

No other specific auditory properties were mentioned as regularly (hence they are grouped under the general “auditory” code). Auditory characteristics described in 250 cases (61%) included references to volume, tone, and speed of speech:

I can hear the *tone and voice inflexions* depending on what the character is saying.

Usually *the tone is distinct*, particular words may be over-pronounced or roll together excitedly. Depending on the situation, if the character becomes stressed or scared for e.g., *the voice changes pitch and speed*…

For 76 cases (18%), the experience of a character did not consist in the quasi-sensory perception of their voice, but rather their manner of speaking, thinking, and experiencing the world – i.e. their *mindstyle* (Fowler, 1977):

It's not only the pitch or sound, but the rhythm of the speech, and the character's *emotion and movement* I get, *it's the whole package*, as if I'm watching a film or in the same space.

With certain favourite books, which I have reread a number of times, I feel I can hear […], the intonation, the way in which they would speak phrases from the text but *also their responses to other situations and problems*.

Visual characteristics were described in 57 cases (14%). In many cases this was described as an immersive, cinematic experience:

Every good book I read that I get in depth with, *plays like a movie*, I don’t really realize it while I’m reading but when I stop I go to find where I’m up to in the book *and I don’t recognise reading the words but I know and have “heard” and “ seen” what has happened*.

Other feelings, such as tactile or olfactory experiences, were also described in a smaller proportion of cases (47 participants; 11%):

Lyra whispering to Will in Pullman's “His Dark Materials”. He describes the loud, busy closeness of her whisper, and *I could hear it and feel it on my neck*.

It feels like I'm sharing the surroundings with the characters or simply experience the landscape*, weather, smells, touch, sounds* etc.

#### 3.3.2 Dynamics

While almost all responses provided some information of the features of vivid reading experiences, not all answers could be coded with a specific dynamic. In those that could be classified in such a way (281 participants; 68%), the dynamics of vivid reading experiences were very varied. The first dynamic coded was *internal blending*, or the process of adapting one’s own inner voice to represent a particular character or narrator. Twenty-two participants (5%) referred to “putting on the voices rather than hearing separate people” or that voices were similar to their own in some way, e.g.:

I think the voice is *a version of mine usually*. I probably *overlay* class, accent etc.

Howard Roark in “The Fountainhead*”* spoke very calmly and cooly but it was *not very different from my own*.

More common was a kind of *external blending*, occurring in 62 cases (15%). In this, participants drew on their experience of others’ voices to simulate a character (often usually a friend, relative, or actor from an adaptation).

The voice of Zooey in Salinger's “Franny and Zooey” always *sounds like a friend of mine who shares many of Zooey's character traits,* including a resonant voice.

If the book has been made into a film then I would tend to hear the actor's voice. Even if I read the book first, *the actors would generally override anything I might originally have imagined*.

Slightly more frequent than both, however, were responses in which the experience of characters’ voices (or thoughts) appeared to break across into new situations, outside of the immediate experience of reading. 77 cases (19%) described this *experiential crossing* of voices:

If I read a book written in first person, *my everyday thoughts are often influenced by the style, tone and vocabulary* of the written work. It's as if the character has started to narrate my world.

Whenever I'm reading a novel I always hear the characters talking even while not reading. *They continue a life between bouts of reading*.

In some cases this was experienced just as a continuation from reading the book; as in one participant’s description of reading Virginia Woolf:

Last February and March, when I was reading “Mrs. Dalloway” and writing a paper on it, I was feeling enveloped by Clarissa Dalloway. I heard her voice or *imagined what her reactions to different situations. I'd walk into a Starbucks and feel her reaction to it* based on what I was writing in my essay on the different selves of this character.

In others this crossing was specifically prompted by familiar or new but similar contexts, i.e. the characters would appear when it would be consistent with their own persona or surroundings:

The character *Hannah Fowler*, from the book of the same name was the voice I heard while walking with my family in the area of Kentucky (USA) where the book took place. I loved the book and *heard her dialogue as I walked through the woods*.[…]

Other than this, participants would often describe their experience of voices and characters in general, non-specific terms that implied an intentional imaginative construction of characters and scenes. This *inner simulation* occurred in 77 cases (19%), e.g.,

I *see the book as a movie* and I am barely aware of the pages. I hear voices, music, and other sounds as described by the author or imagined by me….

I can *visualize and imagine* what the characters sound and look like. The voices, how they express themselves, verbally and non-verbally.

A small number of responses (19 cases; 5%) also described a specific sense of voices “fitting” characters in particular ways, or failing to fit following depiction by an actor or narrator. This *dissonance* of voices could in some cases noticeably interfere with enjoyment of the text. One participant, for example, stated that their experience of voices was:

Vivid enough that when I hear an author speak, I am often surprised how *different they sound than the “narrator” in my head*. It's the same with films; characters often sound “fake” compared to how I have imagined them.

Finally, 24 responses (6%) appeared to describe actual hallucinatory phenomena. Many of these described states in which hallucinations are relatively common, such as in the transition to and from sleep.

I have hypnagogic sleep, so will sometimes hear an *actual voice* whilst falling asleep or awakening, which can be related to what I've been reading before sleep.

## 4 Discussion

The aim of the present study was to survey phenomenological qualities of voices and characters in the experience of readers. Our results indicated that many readers have very vivid experiences of characters’ voices when reading texts, and that this relates to both other vivid everyday experiences (inner speech) and more unusual experiences (auditory hallucination-proneness). However, the features and dynamics of readers’ descriptions of their voices were varied and complex, highlighting more than one way in which readers could be said to “hear” the voice of specific characters. This included quasi-perceptual events across a variety of sensory modalities; personified, intentionally and cognitively rich agents; and characters that both triggered and echoed previous experiences and extra-textual connections.

On the RIQ, the large majority of participants often “heard” character’s voices when reading, with visual and other experiences also occurring frequently. Most found it very easy to imagine characters’ voices during reading, but the vividness of this varied considerably: 1 in 7 participants reported voices that were as vivid as hearing an actual person speak, but double that proportion described either no voices being present or only vague experiences of voice. As such, the experiences reported here should be considered particularly vivid examples of auditory mental imagery, in line with an experiential – and not merely propositional – view of imagery (Kosslyn, Thompson, & Ganis, 2006). This is broadly consistent with inner speech and perceptual simulation accounts of reading (Engelen et al., 2011, Zwaan et al., 2004). However, it would appear to largely fall short of indicating that participants were having literally auditory experiences during reading. This contrasts, for example, with Vilhauer’s (2016) survey, which heavily emphasized such features (e.g.,“An overwhelming majority of [participants] indicated that inner reading voices were audible”, p. 5). We note that the accounts collected in that survey represented a subset of participants who were describing reading experiences after having already referred to “hearing voices”, suggesting that auditory phenomenology (and more specifically, literal hallucinatory experiences or potential psychopathology) may be over-represented in Vilhauer’s (2016) sample.

The relevance of inner speech to this topic is emphasized by the observed relations between the overall vividness of reading experiences and individual differences in the ongoing internal self-talk reported by participants. Regression analysis indicated that participants who reported more elaborate inner speech on the VISQ – being expanded in form, containing dialogue, and the voices of others – were those who also had more vivid experiences of voices and characters during reading. This is a unique demonstration (in terms of empirical research) of the more positive and valuable correlates of phenomenologically diverse inner speech, which in prior studies has correlated with anxiety, depression, low self-esteem, and unusual experiences (McCarthy-Jones & Fernyhough, 2011). Notably, evaluative inner speech – which is linked to low self-esteem (Alderson-Day et al., 2014) – was not significantly associated with reading imagery on the RIQ, once other inner speech and demographic factors were controlled for. It could be that evaluative aspects of inner speech, if present, are more closely linked to negatively-valenced moods and processes (such as rumination; Jones & Fernyhough, 2009), while other features of inner speech may reflect more general imaginative capacities, as indexed by the RIQ used here.

The other factor relating to reading imagery was auditory hallucination-proneness (i.e. LSHS), despite the fact that relatively few participants reported character voices that were as vivid as hearing another person speak. While it is possible that these experiences may nevertheless be linked on a continuum of quasi-perceptual phenomena, the qualitative analysis of readers’ accounts sheds some light on what may link these experiences. Descriptions of vivid auditory imagery were common, but the ways in which this was experienced were highly varied: for some participants this was an intentional, constructive process, for others an automatic immersion, and for others again an experience that appeared to seep out into other, non-reading contexts.

For example, one notable way in which participants talked about their experience of characters was via what we termed “experiential crossing”. We coined this term to refer to instances of characters and voices being experienced outside of the context of reading; a phenomenon that as far as we know has never been studied either in psychological or narratological research. The presence of experiential crossing in nearly a fifth of participants points towards a perfusion of voice- and character-like representations that apparently transgress the boundary between reading and thought. In some cases this was described almost as an echo of prior reading experiences, with auditory imagery re-emerging in a particular context or scenario, but in other accounts it appeared to shape the readers’ style and manner of thinking – as if they themselves had been changed by a character.

Indeed, one discontinuity between experiential crossing and other kinds of dynamic observed in readers’ accounts was the preponderance of fictional characters’ thoughts and feeling over specifically perceptual elements – what was coded as their *mindstyle* (Fowler, 1977, McIntyre and Archer, 2010, Semino, 2007). These readers were engaged with a range of complex cognitive faculties, from beliefs and behavioral patterns to feelings and perceptual biases, with a particular emphasis on the emotional states of characters. All of these become part of the readers’ construction of a fictional consciousness or a “consciousness frame” (Palmer, 2004): a kind of schema for the characters’ worldview and inner experience. This extensive degree of personification that readers process and project into the text can be seen as the counterpart of personifying processes occurring in the writers’ encoding of fictional minds (Taylor, Hodges, & Kohányi, 2003).

Thus, one alternative overlap between vivid reading experiences and hallucination-proneness may lie less in auditory phenomenology and more in the uncontrolled experience of another’s point-of-view; the mindstyle of a character shaping or overlaying the everyday thoughts and feelings of the reader; the social rather than the perceptual. On the one hand this is consistent with accounts of auditory verbal hallucinations that emphasize their intrusive and uncontrollable nature (Badcock, Waters, Maybery, & Michie, 2005) along with their social and agentic characteristics (Wilkinson & Bell, 2016). On the other hand, this would also be consistent with psychological approaches to reading fiction that highlight its interrelation with theory-of-mind (Kidd and Castano, 2013, Mar et al., 2009), and in particular empathy (Mar & Oatley, 2008).

One caveat, however, is that accounts of both mindstyle and crossing were described less like controlled simulations of others’ minds, and more like a habit of thinking or expectation of *what* a character would say in a given situation. If so, this is arguably more similar to the generation and maintenance over time of personality models and agents (Hassabis et al., 2014) than a deliberate and focused act of empathizing or reasoning about another’s mental state (Djikic, Oatley, & Moldoveanu, 2013). One could speculate that the creation of such a ‘consciousness frame’ serves to blur the lines between self and other, leading to the cross-activation of fictional experiences in the reader’s actual world.

The notion of experiential crossing could also be seen as a counterpart to the more widely studied relationship between mental simulation and reader’s previous experiences. If, in experiential crossing, experiences move from the fictional to the real, simulation during reading is activated and supported by the reader’s previous experiences of real-world scenarios – an experiential baggage that has been referred to previously as “repertoire” (Iser, 1980), “encyclopedia” (Dolezel, 2000, Eco, 1984), or “experiential background” (Caracciolo, 2014, Herman, 2004). The creation of such repositories of real-world experiences bears similarities with simulation theories of social cognition that propose the construction of a biographical database on which judgements about other minds are based (Harris, 1992). For example, Green (2004) found that undergraduate participants with personal experience or knowledge of the key themes of a story (a homosexual man attending a university reunion) reported greater transportation into the story and, correspondingly, tended to have beliefs that were consistent with the story ideas. A recent study by Chow et al. (2015) has also found preliminary evidence that past experience of particular scenes and actions directly modulates functional connectivity of visual and motor brain areas during story comprehension.

The role of prior knowledge and experience was most evident in readers’ accounts of internal and external blending of voices, in which participants described drawing on their own voice or others’ to create the voices of characters in the text. In blending, readers seemed to integrate, or “compress” (Turner and Fauconnier & Turner, 2003), their own voice with a textually cued voice (internal blending) or a textual voice with an external source (external blending). The potential differences between internal and external blending remain to be explored: internal blending may depend on personal identification with characters, while external blending, in contrast, could involve an almost unconscious selection of external sources, based on their similarities with the character as described (e.g., in the behavior, beliefs, bodily features, and so on). The fact that – for some participants – the eventual voice could definitely be “right” or “wrong” (as described in cases of dissonance) suggests that representations of characters are quite robust once they are formed. What role character tropes, stereotypes, and prototypes play in this process will be an important avenue of future investigation.

### 4.1 Limitations

It is important to acknowledge some limitations of the present study. First, it is dependent on participants’ self-reports about their general reading experience, which contrasts with experimental approaches that use specific texts and more objective methods of reading engagement (such as eye-tracking). What these data can say about the readers’ experiences of voices and characters is therefore limited by the ability of readers to report on their experiences accurately, and what experiences they choose to describe. Some have argued that readers cannot be relied upon to report on their own experiences accurately and reliably (see Caracciolo & Hurlburt, 2016, for a discussion), but it should be noted that a large body of psycho-narratological work on fiction ultimately depends on participants’ self-reported responses to a story, or other individual differences (e.g., Green, 2004, Mar et al., 2009). Moreover, our intention here was to survey the general reading experience for voices and characters, rather than select a specific text. Individual texts may be particularly evocative of those qualities, but they do not necessarily say anything about how common such experiences are in general reading.

Second, in addressing the question of how readers may be said to “hear” the voices of characters, our study specifically used those terms (voice and character) to probe participants’ experiences. Similarly, our choice of other measures to include reflected this focus, as inner speech and hallucination-proneness are *prima facie* factors that were important to consider. The consequences of this are twofold: (a) it may have led participants to describe predominantly auditory and agent-like experiences over other, more vivid factors (such as visual environments), and (b) it means that other potentially relevant factors, such as transportation and absorption (Kuiken et al., 2004) were not measured here. We would argue that these data are nevertheless informative to understanding the reading experience: voices and characters are prominent in psycholinguistic research (Gerrig, 1993) and narratological approaches to reading (Bortolussi and Dixon, 2003, Herman, 2013, Miall, 2011, Palmer, 2004, Vermeule, 2009), and we note that many of our respondents still described their experience in a much broader fashion (including narrators, visual imagery, and tactile sensations). Nevertheless, it will be important for future phenomenological work to both broaden the scope of inquiry and consider a greater range of individual differences. The role of scene construction, for example, would be expected to play an important role in mental simulation during reading. However, with few exceptions (see “Other”, Table 3) specific references to contextual elements did not feature strongly or explicitly in readers’ descriptions of voice and character (Hassabis & Maguire, 2009).

Finally, it is important to recognize the nature of the sample surveyed. Having been promoted by an international reading festival and a newspaper with global readership, the sample surveyed is much larger and more diverse than most studies of fiction and narrative, which primarily use undergraduate samples. However the sample is still not necessarily representative of a general population, as it is likely skewed towards a well-educated, highly literate readership who may be passionate about fiction (71% reported enjoying reading classics) but engage very little with other genres (only 4% read books on sport). It was also completed by three times as many women as men. As such, the extent to which these results can be generalized to the wider reading population is limited.

Despite these concerns, the present study is to our knowledge one of the only large-sample phenomenological surveys of the reading experience. We argue that the combination of questionnaire items concerning vivid reading experiences in general and more detailed, qualitative analysis of free-text answers offers a broad and varied resource on what it is like to engage with voices and characters in a text. Accounts of the kind collected here pave the way for further analysis of the role played by the reader’s “experiential traces” (Zwaan, 2008) in relation to the simulation of voices and personification. Cases of experiential crossing, on the other hand, suggest a certain kind of rebound, in which fictional agents and worlds are activated in real-life scenarios.

And while our data support the existence of vivid voices and characters in the experience of readers, they ultimately highlight a wide range of quasi-sensory qualities and simulatory dynamics in the reading process. As in debates on mental imagery (Kosslyn et al., 2006), the reading processes described here seemed to be highly varied, ‘experiential’ (Fludernik, 1996), and idiosyncratic. Even if readers’ own accounts are taken as mere indicators of the underlying cognitive and perceptual phenomena, they suggest that the processes by which people produce and experience voices and characters could be very different for different individuals. In this light, the endeavor to describe a typical or normative response to a text becomes perilous, risking an attempt to control the “entropy” of the real experience that individual readers are having (Iser, 2001). The voices and characters of a text are many and various; this would seem to also be true for the reader.

## Figures and Tables

**Table 1:** Demographic information (*n* = 1566).

*Country (top 10 listed)*	*Frequency*	*%*
UK	783	50%
USA	226	14%
Australia	67	4%
Canada	48	3%
Ireland	48	3%
Macedonia	35	2%
New Zealand	32	2%
Germany	21	1%
France	19	1%
India	18	1%

*Education level*	*Frequency*	*%*
Secondary Education	46	3%
GCSE/NVQ	20	1%
A Level	65	4%
Adult/Further Education	132	8%
Undergraduate Degree	643	41%
Masters Degree	482	31%
PhD/Doctoral Degree	171	11%

Which genres do you enjoy reading?*	*Frequency*	*%*
Arts	659	42%
Biography	630	40%
Classics	1106	71%
Crime Fiction	777	50%
General Fiction	1286	82%
General Non-fiction	787	50%
Graphic Novels	368	23%
Historical Fiction	791	51%
History/Politics	735	47%
Poetry	670	43%
Romantic Fiction	303	19%
Science	643	41%
Sci-Fi &Fantasy	857	55%
Sport	60	4%
Travel	440	28%

* Participants could select more than one category.

**Table 2:** Frequencies for reading questionnaire (*n* = 1566).

1. Do you ever hear characters’ voices when you are reading?				
*Never*	*Very Occasionally*	*Some of the Time*	*Most of the Time*	*All of the Time*
166 (11%)	157 (10%)	446 (28%)	468 (30%)	329 (21%)

2. Do you have visual or other sensory experiences when you are reading?				
*Never*	*Very Occasionally*	*Some of the Time*	*Most of the Time*	*All of the Time*
197 (13%)	195 (13%)	425 (27%)	469 (30%)	280 (18%)

3. How easy do you find it to imagine a character’s voice when reading?				
*Very Hard*	*Fairly Hard*	*Neither Easy or Hard*	*Fairly Easy*	*Very Easy*
54 (3%)	104 (7%)	303 (20%)	596 (38%)	508 (2%)

4. How vivid are characters’ voices when you read?				
*No voices present*	*Voices vaguely present*	*Voices with some vivid qualities*	*Voices with lots of vivid qualities*	*As vivid as hearing an actual person*
125(8.0%)	307 (19.6%)	555 (35.4%)	363(23.2%)	216 (13.8%)

5. Do you ever experience the voices of particular characters when not reading?				
*Never*	*Very Occasionally*	*Some of the Time*	*Most of the Time*	*All of the Time*
696(44%)	475 (30%)	339 (22%)	44 (3%)	12 (1%)

**Table 3:** Code definitions for features and dynamics of reading experiences.

	*Description*	*Example*
*Feature codes*		
Character	Specific mention of dominant voice(s) experienced relates to characters’ voices, rather than author/narrator	I usually have a very clear and vivid experience of characters, that I usually imagine who they are, visualise them as if they were in the same room as me and can hear that imagine of the person speak the words the author has given them. […]
Author/Narrator	The dominant voice experienced is that of a narrating figure, identified as either as the flesh-and-blood author, or a fictional narrator within the storyworld	I think (almost exclusively) narratively, so there is always a voice in my head. When I read a book, what I 'hear' is more the voice of the author or my own reading process […]
Accent	Vivid experiences of the characters’ or author’s/narrator’s accents, often related to a specific region and/or country	Most of what I hear isn't necessarily their 'voice,' but the accent I imagine them to have
Mindstyle	A worldview of a specific individual (narrator or character), which is constructed, experienced and reported in terms of their cognitive perspective (beliefs, emotions, biases, and so on) on the storyworld	I imagine the voice, how it resonates, imagine the person, what they are thinking, what they would think of a particular issue if I discussed it with them. What would they say? Etc
Auditory	Specific reference to quasi-sensory auditory phenomena, either related to the voices of characters or to those of authors/narrators (e.g., tone, volume) but not related to accent	It's less voices, unless the character is particularly strong, Terry Pratchet is good for those, and more a general image of them and particularly their surroundings, so sounds surrounding them, almost like sound effects, or a soundscape
Visual	A specific sense of quasi-sensory visual phenomena, either as a bare series of snapshot images or a more rich “spectatorial” perspective on character or scene	I don't have particularly vivid experiences hearing voices, but I stop seeing the words I am reading, and see what is happening in the story instead. […]
Other	References to other specific sensory or experiential qualities, including tactile and bodily responses	It feels like I'm sharing the surroundings w the characters or simply experience the landscape, weather, smells, touch, sounds, etc.

*Dynamic codes*		
Internal blending	Voices experienced are a combination or mixture of the reader's own inner voice with the qualities of the voices described in the text (i.e. accents, pitch, tones, genre)	I think the voice is a version of mine usually. I probably overlay class, accent, etc.
External blending	Voices experienced are a combination or mixture of the reader's own voice with voices previously heard in the external world (e.g., voices of personal acquaintances or voices of actors in a movie)	Usually if I have watched the film version before reading, the voices will be those of the actors when I read the book […]
Experiential crossing	Voices are experienced outside of the immediate context of reading, i.e. they seem to *cross* the boundary of the storyworlds and accompany or “stay with” the reader in real-world situations	[…] If the 'voice' of a good book gets into my head, it can seep into my own experience of the world and I find myself thinking in that voice, as that character, while carrying out normal activities
Inner simulation	A feeling of *actively* needing to imagine and shape full-blown fictional scenarios in order to make sense of the text (as compared to passively 'hearing' or 'seeing' a character)	I form a picture of what the character looks and sounds like in my head, and when reading about them in the book or even thinking back on the character I can hear them speak in the accent and voice of the character formed in my imagination
Dissonance	When readers experience a specific feeling of mismatch or clash between how they imagined a character’s voice and its depiction (such as when watching a movie adaptation of a novel)	[…] I usually get really disappointed watching films made of books because I have a very clear idea of the world in which the characters live, the voices they have, their appearance and mannerisms and a film version never matches this. It's almost like the book is more vivid for me than watching the film
Actual hallucinations	Descriptions of voices that appear to be literally hallucinatory and may or may not be related to reading (e.g., accounts of psychotic episodes, sleep-related hallucinations	As a child once when I had a high fever and was hallucinating, the hallucination seemed to be mixed up with characters in a book I was reading

**Table 4:** Hierarchical regression analysis for reading imagery questionnaire (RIQ) results (*n* = 1513).

	*B*	*SE B*	*Beta*	*t*	*p*	*C.I. (95%)*		*F*	*df*	*p*	*adj. R2*
Age	−0.01	.01	−0.03	−1.03	.30	−0.02	.01	4.54	3, 1509	<0.01	.01
Gender	.66	.25	.07	2.63	.01	.17	1.15				
Education	−0.22	.09	−0.06	−2.45	.01	−0.39	−0.04				
Age	.00	.01	.01	.59	.56	−0.01	.02	43.06	8, 1504	< 0.001	.18
Gender	.41	.23	.04	1.77	.08	−0.04	.86				
Education	−0.09	.08	−0.03	−1.07	.28	−0.25	.07				
Auditory Hallucination-Proneness (LSHS)	.17	.04	.11	4.45	< 0.001	.09	.24				
Dialogic IS (VISQ)	.09	.02	.10	3.66	< 0.001	.04	.13				
Evaluative IS (VISQ)	.00	.03	.00	.08	.94	−0.05	.06				
Other People IS (VISQ)	.17	.02	.30	11.37	< 0.001	.14	.20				
Condensed IS (VISQ)	−0.06	.02	−0.09	−3.81	< 0.001	−0.10	−0.03				

**Abbreviations: IS** Inner Speech; **LSHS** Launay-Slade Hallucination Scale; **VISQ** Varieties of Inner Speech Questionnaire.

## Footnotes

2 Nine participants described their gender as “Other”. For parsimony, gender was modeled as a single categorical variable (male/female) with these 9 participants excluded, resulting in a sample of *n* = 1513 for the regression analysis.

3 As a further check on the RIQ results, we reran the correlation and regression analyses using a version of the VISQ adapted for frequency rather than agreement (the VISQ-2). Instead of rating statements based on agreement, participants were asked to rate how often certain experiences occurred on a scale from 1 (Never) to 7 (Almost Always). This produced almost identical results for each bivariate correlation with RIQ (all *r* within ±0.03 of the above). For the regression analysis, all of the original significant predictors were identified (with very similar beta values) but in addition Gender was retained for the final model (*β* = 0.050, t = 2.12, *p* = 0.03, 95% *C.I.* = 0.037–0.943). The direction of the coefficient indicated that women were slightly more likely than men to report vivid reading imagery. The updated VISQ-2 is currently in development and is available on request from the corresponding author.
